# Using a full thickness bioengineered human skin equivalent as a model for radiation biology research

**DOI:** 10.1038/s41598-025-17153-4

**Published:** 2025-10-06

**Authors:** Geraldine Vitry, Jerry Angdisen, Megan A. Sawant, Pauline Arriaga, Shawn Irgen-Gioro, Paul Peshette, Daniel C. Vuong, Peter Ilhardt, Jacques Fehr, Bartosz Cwikla, Brian Ponnaiya, Jamie L. Inman, Antoine M. Snijders, Shazia Hamid, David Caballero-Lima, Guy Garty, Karyn Apfeldorf, Evagelia C. Laiakis

**Affiliations:** 1 Department of Oncology, Lombardi Comprehensive Cancer Center, Georgetown University Medical Center, Washington, DC USA; 2 Data Analytics, Areté Associates, Northridge, CA USA; 3 Currently at: Amentum, NASA Johnson Space Center, Houston, TX USA; 4 Radiological Research Accelerator Facility, Columbia University, Irvington, NY USA; 5 Labskin LTD, York, UK; 6 Center for Radiological Research, Columbia University Irving Medical Center, New York, NY USA; 7 Lawrence Berkeley National Laboratory, 1 Cyclotron Road; MS 977-162, Berkeley, CA USA; 8 Currently at: Biosciences and Biotechnology Division, Lawrence Livermore National Laboratory, Livermore, CA USA; 9 ATCC, 10801 University Boulevard Manassas, Manassas, VA USA; 10 Microsapient, Freelance, London, UK; 11 Department of Biochemistry and Molecular & Cellular Biology, Georgetown University Medical Center, Washington, DC USA; 12 Department of Radiation Medicine, Georgetown University Medical Center, Washington, DC USA; 13 Center for Metabolomic Studies, Georgetown University, Washington, DC USA

**Keywords:** Radiation biology, Bioengineered skin, Skin microbiome, Multi-omics, Biodosimetry, Mass spectrometry, Metabolomics, Preclinical research, Biomarkers

## Abstract

Radiation exposure from radiological or nuclear events, medical treatments, or spaceflight poses significant health risks, yet human-specific models to investigate radiation effects on skin remain limited. This study establishes a novel in vitro platform using a full-thickness bioengineered human skin equivalent colonized with natural mixed human microbiota (coHSEs) to assess radiation-induced biological responses. We exposed coHSEs to acute doses of up to 4 Gy with x-rays and evaluated their viability, structural integrity, and molecular responses over 25 days. The coHSE model demonstrated sustained viability without dose-dependent opportunistic microbial overgrowth when procedural optimizations were applied. Radiation-induced epidermal remodeling did not compromise tissue architecture or swabbing-based sample collection. Cell proliferation analyses revealed dose- and time-dependent dynamics, with consistent dermal cell density maintained across radiation doses. Comparative multi-omic analyses, including untargeted metabolomics, targeted lipidomics, and 16 S metagenomics, revealed conserved metabolic and microbial responses to radiation in both coHSEs and skin from irradiated mice. Enriched pathways such as arachidonic acid and fatty acid metabolism, along with shifts in microbial taxa including *Lachnospiraceae*, support the translational relevance of the coHSE model. This system offers a scalable, ethical, and physiologically relevant platform for radiation biology, biodosimetry, and therapeutic development, advancing terrestrial health research with promising application for space research.

**Supplementary Information:**

The online version contains supplementary material available at 10.1038/s41598-025-17153-4.

## Introduction

Radiation-producing technologies, including nuclear energy and nuclear medicine, have become essential to modern life, increasing the risk of human exposure to low-dose radiation. Radiological or nuclear accidents or malicious uses, such as detonation of an improvised nuclear device (IND), can disperse high-energy radiation and radionuclides over vast distances. Simultaneously, human expansion into space due to planetary programs raises exposure to space radiation. Consequently, radiation biology research is crucial for understanding the effects on exposed populations, including nuclear workers, radiologists, disaster victims, emergency responders, radiotherapy patients, aircrew members, astronauts, and space tourists, while aiding in the development of tailored healthcare and preventive measures.

The skin, as the first layer of the human body exposed to radiation, offers accessibility for non-invasive and real-time monitoring. Its multilayered structure includes the hypodermis, dermis, epidermis, and microbiome, which mainly resides on the stratum corneum and consists of over 200 bacterial genera. Although radiation-induced skin injuries have been histopathologically characterized, underlying molecular mechanisms and biomarkers remain insufficiently explored^1,2^. The skin, involved in thermoregulation and blood pressure control, receives substantial blood flow carrying circulating signals making it a potential site for detecting systemic radiation responses. Skin conditions have been linked to cancers, liver diseases, and cardiovascular diseases, with prognostic biomarkers identified^3–9^. Microbiome shifts have also been correlated with clinical outcomes, including increased radioprotective bacterial species in the^6,10–17^. Therefore, skin molecular and microbial changes represent promising avenues for radiation exposure monitoring and proactive medical countermeasures.

Traditional in vivo and in *vitro* models, including mouse models and 2D skin cell cultures as gold standard, have limitations. The multicellular and layering complexity (e.g. immune cells, fibroblasts, melanocytes, keratinocytes, glands, and sensory neurons) ensuring the skin function as physical barrier, and thermoregulation, blood pressure regulation, and environmental sensory interface are not recapitulated by 2D skin cells models. Meanwhile, the mouse skin differs significantly from human skin, which contains 6–32 epidermal layers and unique features such as rete ridges absent in rodents^18^. The murine dermis also has a panniculus carnosus layer, affecting wound healing mechanisms compared to humans^19,20^. Additionally, the mouse microbiome, shaped by highly controlled laboratory conditions and standardization, do not fully represent human microbiome diversity whose composition depends on environmental conditions as well as maternal and birth determinants. Advances in both mass spectrometry-based -omics studies and 3D in vitro models provide us with enhanced capabilities in capturing and understanding the molecular mechanisms underlying the response to radiation in humans.

Bioengineered human skin equivalents (HSEs) can more accurately mimic skin structure and paracrine dynamics while allowing long-term studies. Some models, such as Labskin, support microbial colonization^21–23^. Colonizing HSEs with natural microbiota remains challenging due to potential skin barrier disruption and opportunistic microbial overgrowth. Most studies inoculate only a few microbial strains instead of complete mixed communities^24–26^. However, with a population density ranging from 1 million to 1 billion microorganisms per cm^3^the skin microbiome outnumbers the human cell and genome, thus holding the potential to significantly outpace the human contribution to the metabolism and phenotype^27–30^. As such, skin models with incomplete or lacking microbiota, are likely failing capturing the physiological reality. For instance, the colonization of an HSE model with a complete microbiome has been found to be more beneficial to the skin barrier than with a single strain alone^31,32^. Additionally, Pannkuk et al. demonstrated that antibiotic microbiome depletion in mouse gut significantly attenuated urine and serum metabolomic profiles after radiation exposure compared to non-depleted mice^33^. Nonetheless, a radiation signal was persistent reinforcing the relevance of microbiome analyses in biodosimetry. While metabolomic responses to radiation have been explored using bioengineered HSEs, no study has yet examined irradiated HSEs colonized with natural human microbiota, hindering part of the complete picture of the biological response of the human skin to radiation and potential biomarkers.

The aim of this study was to evaluate the viability of exposing colonized human skin equivalents (coHSE) to radiation and their relevance as a radiation biology model. We selected X-rays (320 kVp) as the radiation type due to their established use in radiobiology and their biological comparability to gamma rays, the dominant photon radiation encountered in nuclear accidents, radiotherapy, and spaceflight. X-rays and gamma rays are both high-energy photons and share similar linear energy transfer (LET) and dose deposition characteristics, making X-rays a practical and physiologically relevant surrogate for controlled in vitro experimentation. The 1–4 Gy dose range was chosen to reflect clinically relevant exposures, such as those used in radiotherapy (up to 2 Gy/fraction), and sub-acute doses associated with occupational, accidental, or spaceflight scenarios, which typically range from 0.1 to 1 Gy for cumulative whole-body exposure, and up to several Gy for localized exposure. This design enabled us to evaluate the viability and molecular responsiveness of the coHSE model under realistic and translationally relevant photon exposure conditions. We analyzed microbial overgrowth after inoculation, tissue quality post-swabbing, and histological integrity following up to 4 Gy x-ray exposure. We further evaluated cell survival via immunofluorescence, western blot analysis of proliferation markers, and cell density measurements. Lastly, we characterized molecular responses in coHSEs versus skin from irradiated mice using untargeted metabolomics, targeted lipidomics, and microbiome sequencing, including alpha and beta diversity analyses. To our knowledge, this is the first study to evaluate long-term molecular and microbial responses to radiation in a human skin equivalent colonized with natural microbiota, offering a more physiologically relevant in vitro model than traditional 2D cultures or uncolonized HSEs.

## Results

### Radiation exposure does not affect opportunistic microbial overgrowth occurrence in HSE inoculated with mixed microbial communities

Experiments were conducted with two sequential batches of the Labskin HSE products, to allow for the possibility of process optimizations during the study. Labskin HSE were inoculated 1 day after their arrival and irradiated the day after, corresponding to air/liquid interface (ALI) growing day 15 and 16 respectively (Supplemental Fig. 1).

**Fig. 1:**
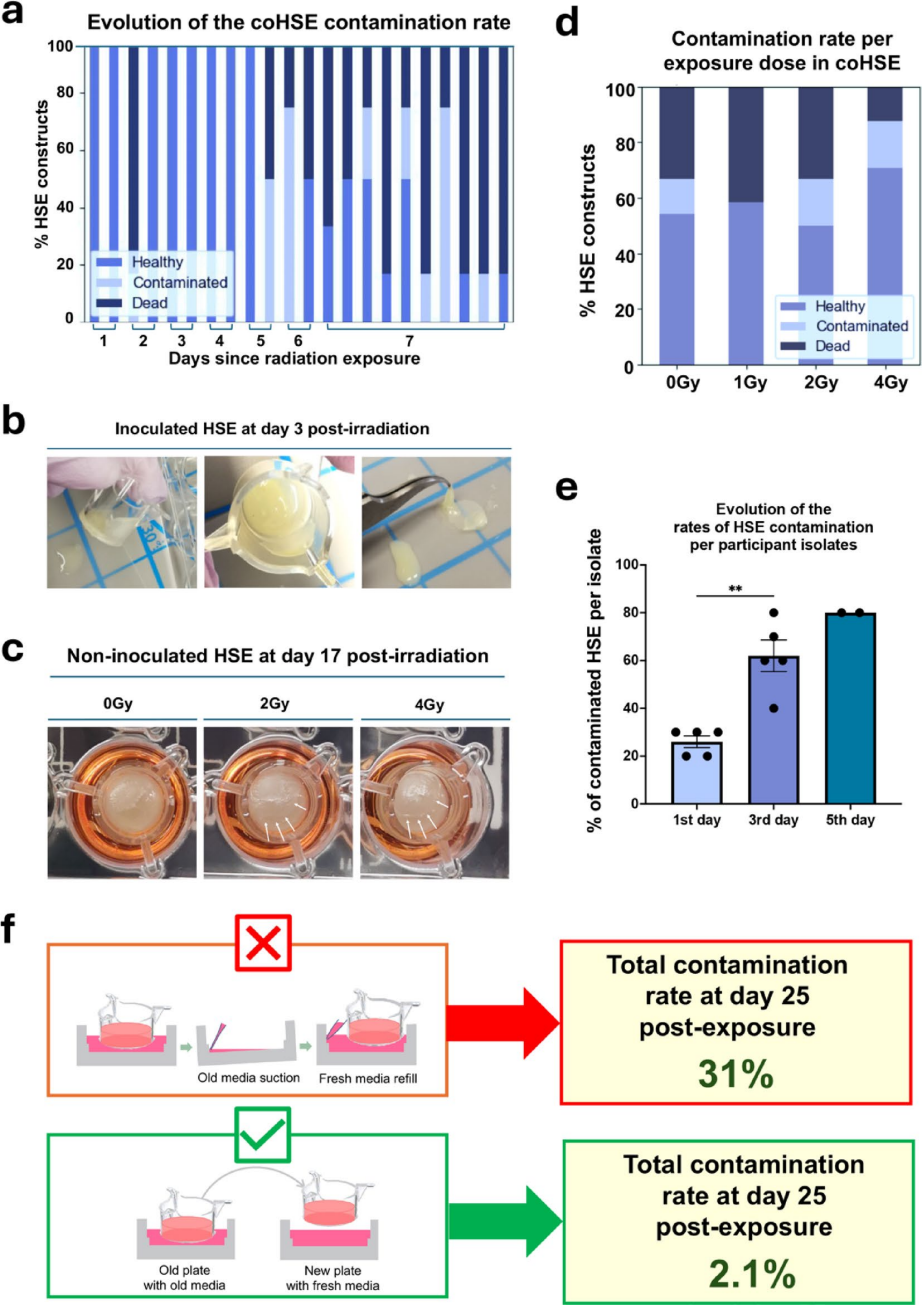
Effects of the combination of radiation exposure and inoculation of mixed microbial communities on human skin equivalents (HSE) culture. **a**) Percentage of contaminated HSE constructs (*n* = 180 constructs) per participant isolates (*n* = 18 participants) over time (*n* = 4 constructs per isolates/dose/time point), **b**) Photos of highly contaminated inoculated HSE 3 days post x-ray exposure, **c**) Photos of non-inoculated HSE at day 17 post x-ray exposure, **d**) Percentage of inoculated HSE contamination per exposure dose (*n* = 24 constructs per exposure dose), **e**) Percentage of contaminated HSE constructs per participant isolates (*n* = 5 isolates) the 1 st day of opportunistic microbial overgrowth observation, the 3nd day after the first opportunistic microbial overgrowth day observation, and the 5th day, independently of the time since exposure and inoculation, (each dot represent a participant isolate), and **f**) Schema of simple modifications introduced in colonized HSE culture procedures that radically improved contamination rates.

Irradiation experiments had never been performed on inoculated HSE before, therefore we investigated the combination of the 2 stresses on the physiology of HSE. Bacterial penetration through the epidermal and dermal layers can result in opportunistic overgrowth and contamination. In addition to suboptimal procedural stringency, aggressive handling during inoculation, such as vigorous circular spreading of the inoculum, can compromise the structural integrity and barrier function of the bioengineered skin, facilitating microbial infiltration. In our first experimental batch, contamination rates increased over time, correlating with rising HSE mortality. By day 7, 61% (37 out of 60) of the remaining wells exhibited signs of contamination (Fig. 1a and b). The experiment with inoculum + irradiation lasted 7 days, a reduced life span possibly due to the combination of the two stressors (irradiation and microbial inoculation). At the end of the 1 st experiment, ~ 35% (63/180) of the total number of Labskin HSE presented with contamination. Non-inoculated HSE were also irradiated and cultured together with inoculated HSE plates and were maintained in culture for 25 days post exposure with no sign of opportunistic microbial overgrowth. Figure 1c, shows pictures of non-inoculated HSE at day 17 post-irradiation. An asymmetry was noticed of the surface structure in the exposed groups not visible in the non-exposed group (white arrow), suggesting that radiation exposure may induce tissue remodeling and change in properties such as permeability. However, the rate of opportunistic microbial overgrowth and mortality together varied between 30% and 40% with no dose dependent pattern, and with 4 Gy having the lowest rate (Fig. 1d). Conversely, the progression of the rate of HSE construct contamination per participant isolate over time is variable (Fig. 1e). On the first day of observable signs of opportunistic microbial overgrowth, ~ 20% of the overall constructs were affected. However, on the third day after the first observation, the rate varied from 40 to 80% among the participant isolates, indicating that certain isolates may contain more virulent strains than others. The subsequential batch experiment, included changing the feeding procedure for construct transfers into new plates filled with fresh media instead of the suction/refill method (Fig. 1f). This reduced opportunistic microbial overgrowth by preventing potential cross contamination between wells, yielding a contamination rate at 2.1% of total HSE constructs and allowing experimentation to proceed to 25 days post radiation exposure.

### The epidermis quality is homogenous in all irradiated CoHSEs groups

**Fig. 2:**
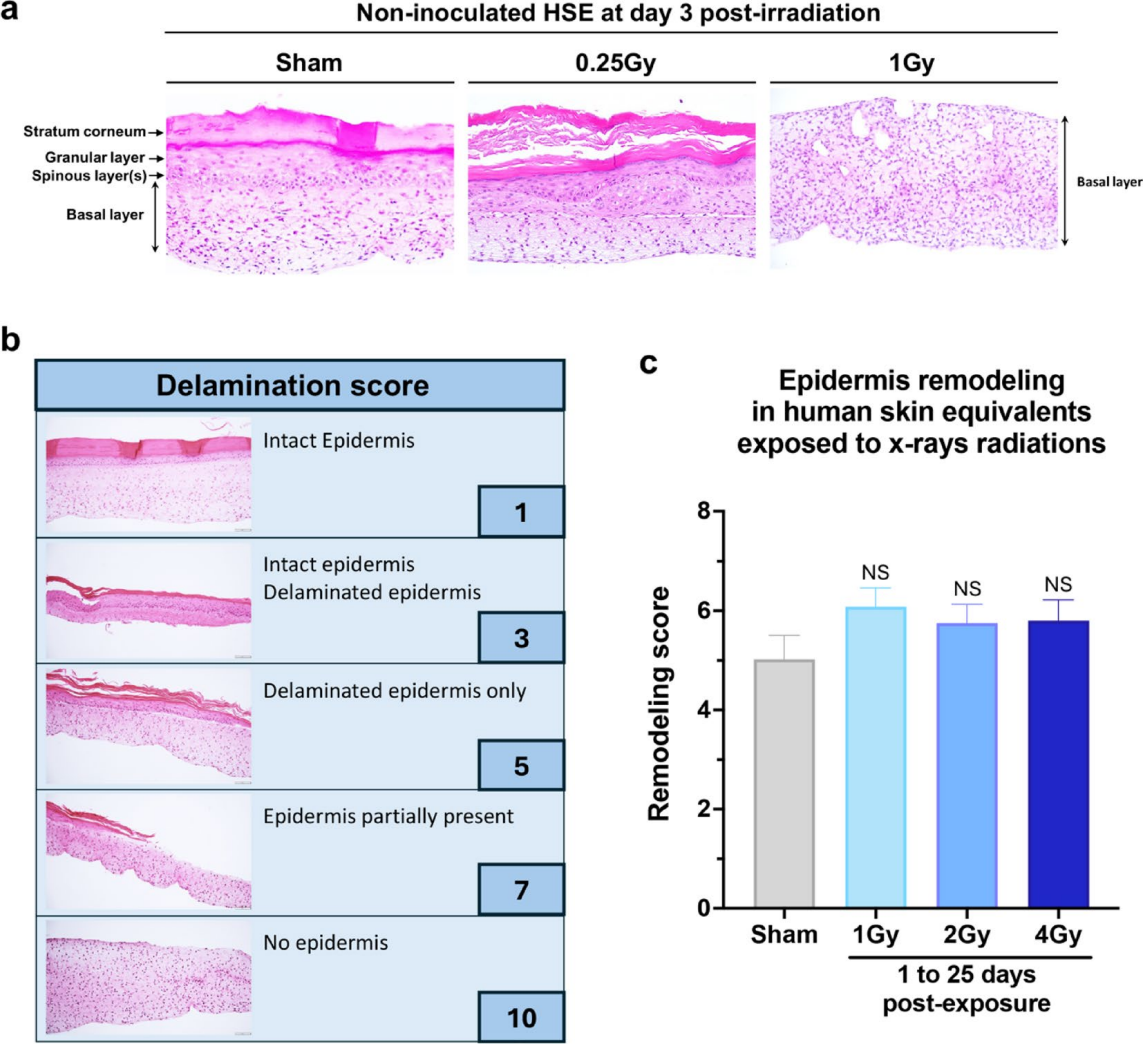
Effects of radiation exposure on coHSE epidermis structure. **a**) Hematoxylin & Eosin staining of non-inoculated HSE sections from non-exposed (sham) and exposed (0.25 Gy and 1 Gy) groups 3 days post-exposure in preliminary studies, **b**) Epidermis Remodeling classification and score, and c) Remodeling score in non-exposed (sham, *n* = 38) and 1 Gy (*n* = 36), 2 Gy (*n* = 45) and 4 Gy (*n* = 46) exposed coHSE groups up to 25 days post-exposure (*n* = 4–7 constructs per time point per exposure dose).

Figure 2a shows H&E stained tissue sections from non-exposed and exposed non-inoculated HSE groups in a preliminary study at low doses. HSE exposed groups (0.25 and 1 Gy) displayed a trend of increased delamination or even complete loss of the epidermis, however due to the small sample size (*N* = 2–3 per group) statistical analysis was not feasible. Radiation exposure is associated with desquamation, which is the shedding of the outer layers of the skin^34,35^. As such, sample swab collection in exposed HSE may be biased by desquamation as it would affect the type and the quantity of material available for the swabbing. We measured the delamination level in exposed versus non-exposed coHSE section stained with H&E using a scoring system (Fig. 2b). Delamination scores from 1 to 10 were assigned according to the extent of the delamination in the epidermis, with 1 corresponding to no delamination (intact epidermis) and 10 to the complete loss of epidermis (no epidermis). The quantification of the scores (Fig. 2c) shows that there are no significant differences in the delamination level between doses indicating that swab sample content should not be biased by radiation induced tissue injury (0 Gy *n* = 8, 1 Gy *n* = 6, 2 Gy *n* = 9, or 4 Gy *n* = 6).

### Irradiated CoHSEs sustain survival capacity over time

The difference in cell proliferation and the total number of cells in a tissue is a good indicator of cell survival and can vary among tissue components, area or layers. HSE were shipped on day 12 of air/liquid interface (ALI) growing with a cell density of 1.30 × 10^2^ mm^−2^ in the epidermis and 1.15 10^2^ mm^−2^ in the dermis (Supplemental Figs. 1 A and 1B). Twenty-four hours post-inoculation, or ALI day 16, HSE cell density was ~ 10 times increased in both the epidermis and the dermis (10^3^mm^−2^) compared to the day of shipping count (ALI 12), indicating a maintained proliferation activity in the 2 layers (Supplemental Fig. 1 C).

**Fig. 3:**
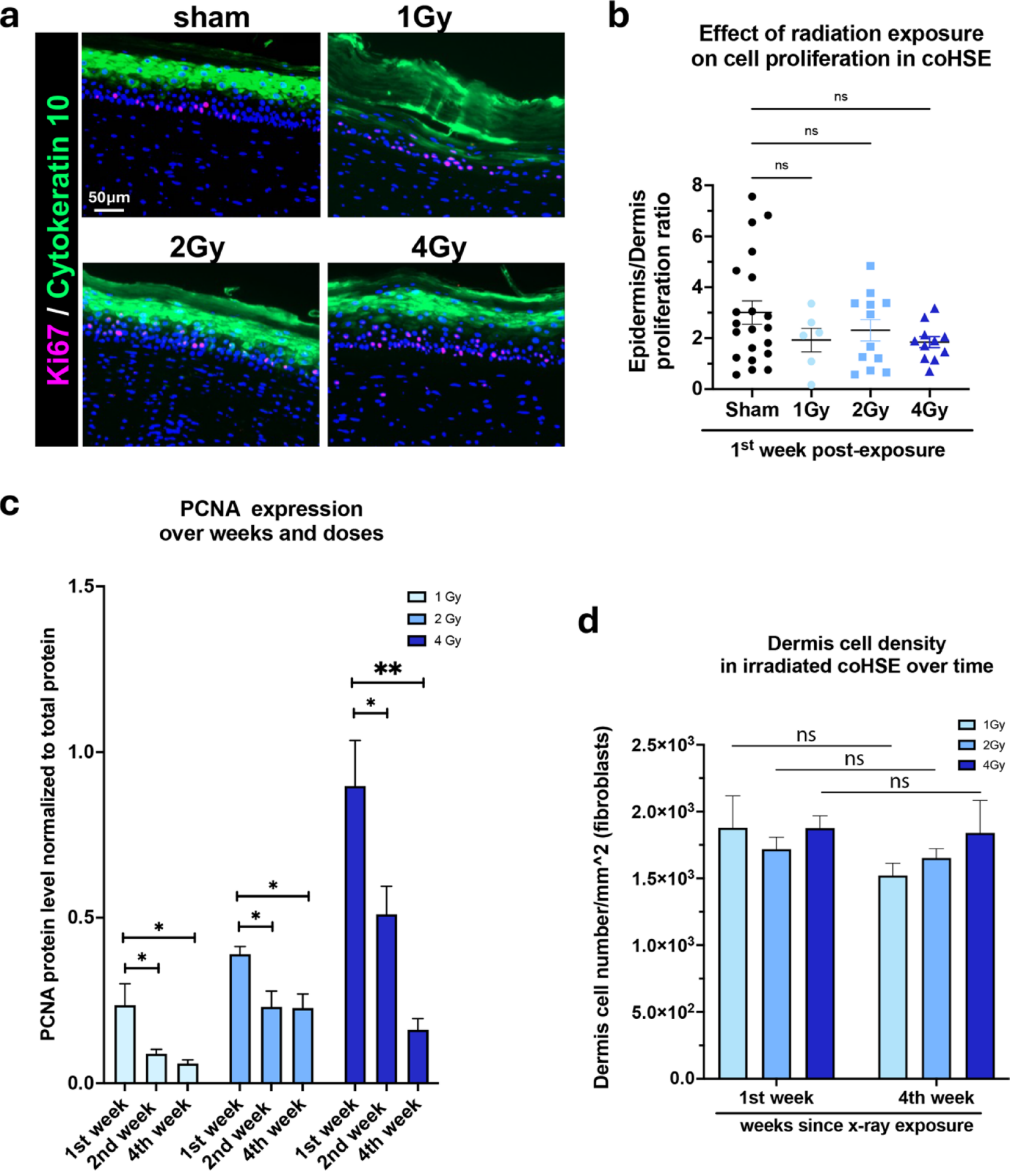
Effects of radiation exposure on colonized human skin equivalents (coHSE) cell proliferation. **a**) Representative images of Ki67 immunofluorescence in coHSE sections from non-exposed (sham), and x-ray-exposed (1 Gy, 2 Gy, and 4 Gy) groups within the first week after radiation exposure, **b**) associated quantification graph showing the epidermis/dermis proliferation ratio (the percentage of Ki67 positive cells in epidermis over the percentage of Ki67 positive cells in dermis), **c**) Protein level expression of PCNA normalized to total protein (Amidoblack) measured by western blot analysis, in full thickness coHSE the 1 st, 2nd, and 4th week post exposure to x-rays at 1 Gy (*n* = 5 or 6 constructs/week), 2 Gy (*n* = 5 or 6 constructs/week) and 4 Gy (*n* = 5 or 6 constructs/week), and d) Dermis cell density (number of cell per mm^2^ in all exposed coHSE group the 1 st week (*n* = 8–12 constructs per dose) compared to the 4th week (*n* = 4–7 constructs per dose). Full images of blots are available in Supplemental Figs. 4 & 5.

We measured the percentage of Ki67- positive cells by immunofluorescence to evaluate the proliferation rate in both the dermis and the epidermis (partially highlighted in green with the immunofluorescence of the cytokeratin 10, “K10”) of irradiated coHSE during the first week post exposure (Fig. 3a). The epidermis/dermis proliferation ratio (percentage of Ki67 positive cells in epidermis over the percentage of Ki67 positive cells in dermis) tended to be higher in the non-exposed coHSE group than in the exposed groups, however no significant differences were found between exposed groups, suggesting that cell proliferation is evenly affected by radiation x-ray exposure in both the dermis and epidermis (Fig. 3b). However, the cell proliferation trend was non-linear with the time since exposure within the first week and was not dose dependent in the dermis of exposed coHSE (Supplemental Fig. 1 d). The 2 Gy and 4 Gy coHSE groups displayed a similar pattern of cell proliferation rate (11–13% of proliferating cells) with a similar trend of decrease the 1 st day post exposure followed by an increase the 3rd day, reaching a close proliferation rate the 7th day (8–9%). Surprisingly, the 1 Gy group had the lowest cell proliferation rate the 1 st day post exposure (3%), but had the highest increase trend of the cell proliferation rate between the 3rd and the 7th day post exposure (reaching 25%). The 1 Gy group was also found with the lowest protein levels of the proliferation marker PCNA during the 1 st week, and over 4 weeks post radiation exposure (Fig. 3c). A clear dose response effect on the expression of PCNA was maintained over 2 weeks post irradiation; unexpectedly, PCNA protein levels increased with dose. Conversely, the proliferation rate decreased with time at each dose, which could potentially reflect the confluence limit. Nonetheless, cell density in the dermis remained around 2 × 10^3^ cell.mm^−2^ over the 4 weeks in all exposed groups, indicating a similar development of the dermis between doses up to 4 Gy (Fig. 3d).

All together, these results demonstrate that the present coHSE model is viable for studying the biological effects of radiation exposure at doses relevant to various exposure scenarios (1–4 Gy) and comparable to x-ray energies. Additionally, the model remains viable over 4 weeks post-exposure enabling extended experimental design.

### Molecular responses to x-ray exposure between CoHSE and mouse skin

**Fig. 4:**
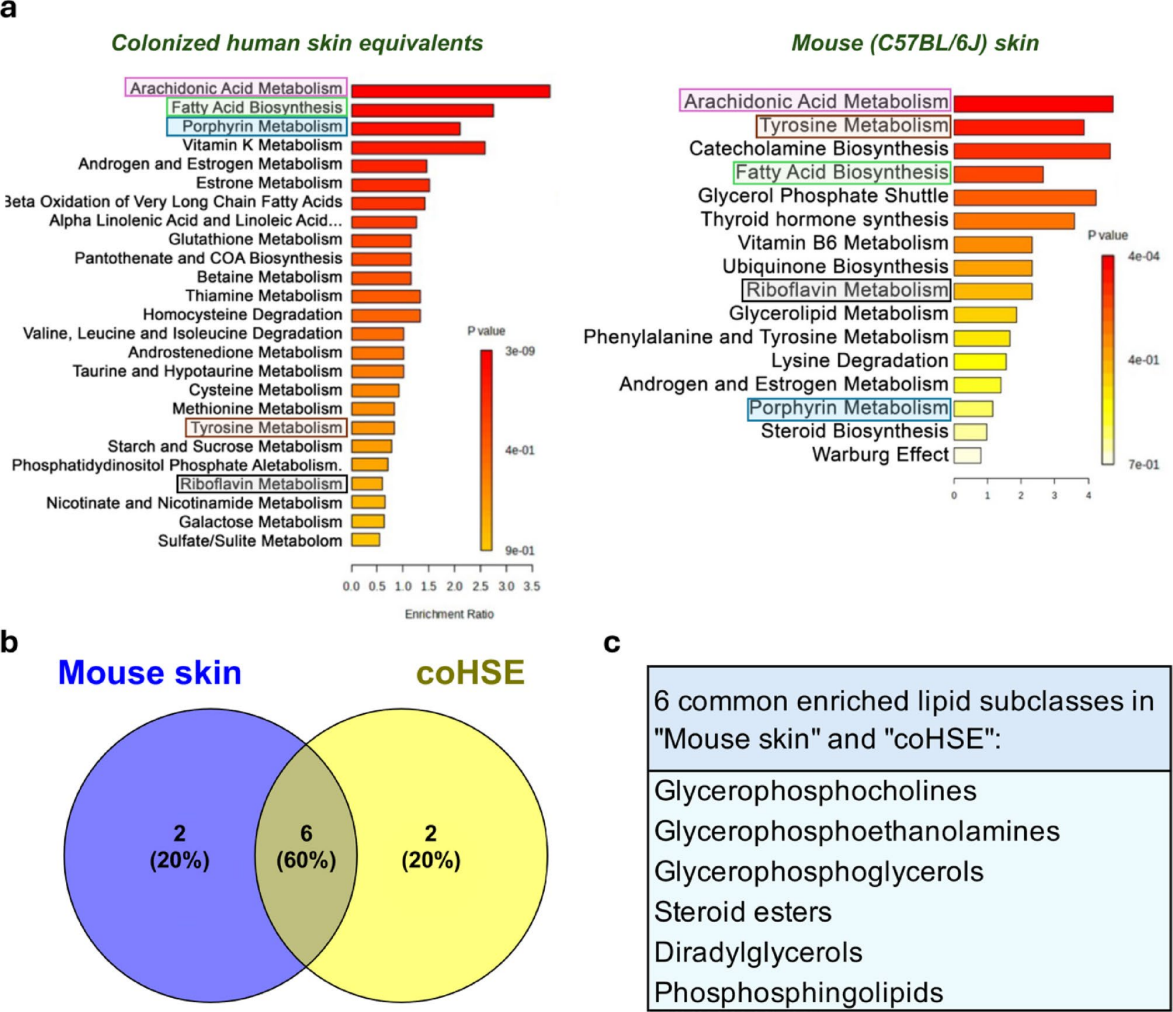
Molecular response to radiation in coHSE vs. in mouse skin. **a**) Over representation analysis (ORA) of metabolic pathways at 1, 2, and 4 Gy in coHSE (*n* = 6 per group) and in mouse skin (*n* = 6 per group) using the small molecule pathway data base (SMPDB) library on the MetaboAnalyst 6.0 online software, based on putatively identified sPLSDA top features (VIP score > 1), **b**) Venn diagram of lipid subclass ORA of lipids with a VIP score > 1 (partial least square discriminant analysis, PLS-DA) found in common within all exposure doses (1, 2 and 4 Gy) in coHSE and mouse skin using MetaboAnalyst 6.0 online software, and c) The list of common enriched lipid subclasses between coHSE and the mouse skin at all doses (1, 2, and 4 Gy).

The human and mouse skin share a common organizational plan. However, the structural and cellular differences as well as environmental conditioning may be reflected in their molecular and microbial phenotype. Figure 4a shows the over representation analysis (ORA) of the top metabolomic features (sPLSDA VIP score > 1) that were found in common in all exposed (1, 2 and 4 Gy) coHSE groups (left) or mouse groups (right) based on putative identification, and using the small molecule pathways database library (SMPDB)^36^. Among these pathways, 5 were enriched in both coHSE and mouse skin including porphyrin, tyrosine, and riboflavin metabolism, as well as certain lipid metabolism pathways such as arachidonic acid metabolism and fatty acid metabolism. ORA of lipidomic data (Fig. 4b) shows that the mouse skin and coHSE share about 80%of lipid subclasses commonly enriched in response to radiation according to the lipid subclass library containing 1250 sub chemical class metabolite sets or lipid sets.

### Microbial responses to x-ray exposure between CoHSE and mouse skin

**Fig. 5:**
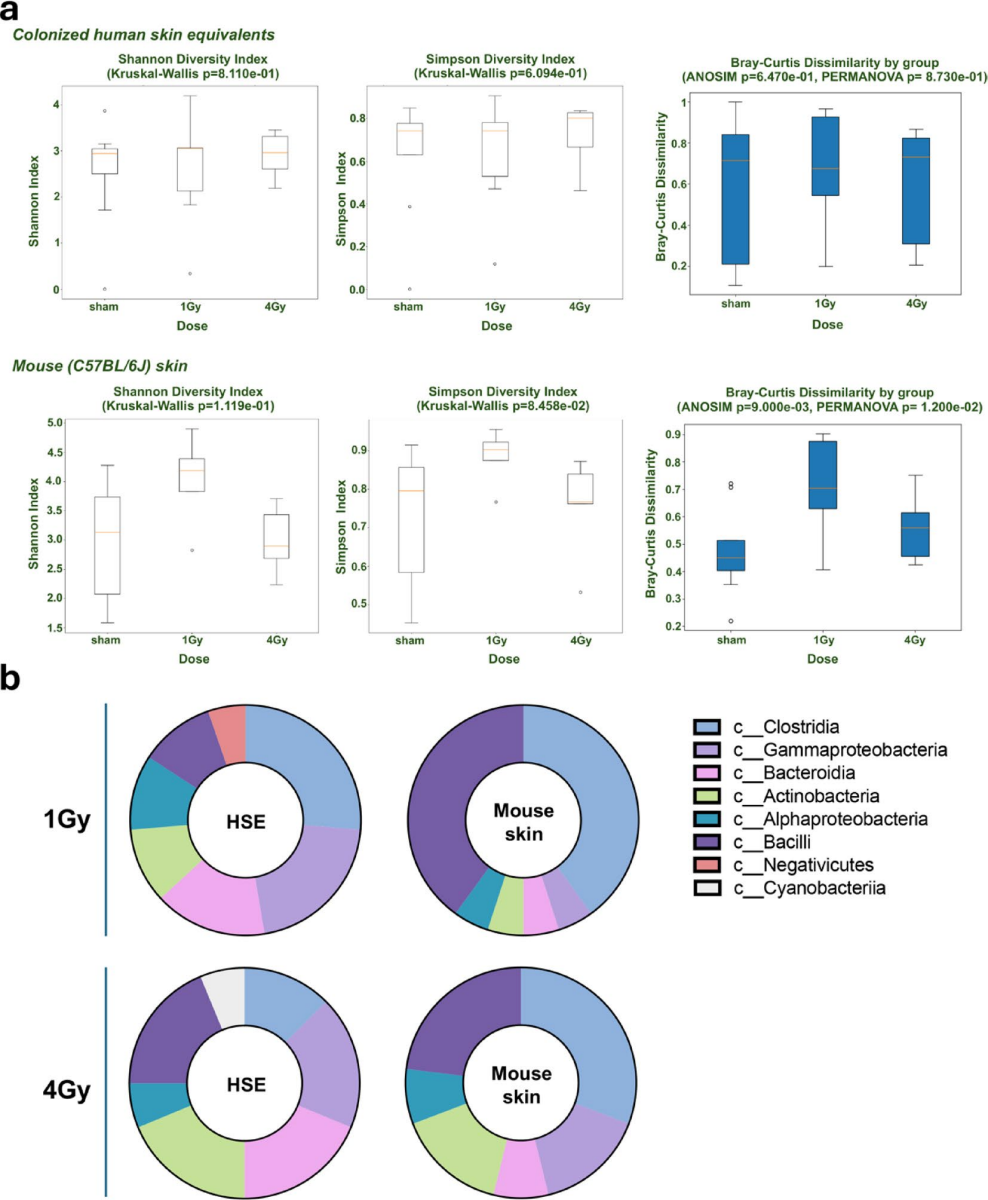
Microbial changes in response to radiation in coHSE vs. in mouse skin. **a**) Alpha Diversity (Shannon and Simpson Indexes) and Beta Diversity (Bray-Curtis Dissimilarity) in irradiated coHSE (*n* = 18) and mouse skin (*n* = 18) at 1 Gy (*n* = 6) and 4 Gy (*n* = 6) compared to sham (*n* = 6), and **b**) Significantly impacted microbial classes (metagenomic 16 S) at 1 Gy and 4 Gy in HSE and mouse skin (p-value < 0.05).

The Shannon Diversity Index value of coHSE was consistent across all three exposed groups (sham, 1, and 4 Gy), with a mean value of approximately 2, indicating that species richness remained stable across different radiation conditions (Fig. 5a upper panels). In contrast, the mean Simpson Diversity Index was highest in coHSE samples at 4 Gy, though this difference was not statistically significant (Kruskal-Wallis p-value > 0.05). This suggests a shift in community composition at 4 Gy, where certain species may become more dominant in an uneven distribution, despite the stable species richness indicated by the Shannon Index. The Bray-Curtis Dissimilarity for coHSE across all three radiation doses had a mean value of 0.617, indicating high variability in community composition. However, both ANOSIM (*R* = −0.0408, p-value = 0.647) and PERMANOVA (Pseudo-F = 0.4059, p-value = 0.873) confirmed that these differences were not statistically significant, suggesting that radiation exposure did not significantly impact beta diversity in coHSEs (Supplemental Fig. 3).

In the mouse skin samples, the 1 Gy samples showed the highest mean Shannon Diversity Index (0.884 ± 0.072), followed by sham and 4 Gy (Fig. 5a bottom panels). This pattern suggests that moderate radiation exposure (1 Gy) promotes a more diverse and evenly distributed microbial community compared to both untreated (sham) and high-dose (4 Gy) conditions. The lowest Shannon Index at 4 Gy (2.992 ± 0.587) indicates that higher radiation doses disrupt microbial diversity, potentially favoring a less balanced community composition. The Simpson Diversity Index followed a similar trend, with 1 Gy samples having the highest index.

PERMANOVA results (Pseudo-F = 2.1378, p-value = 0.012) suggest that the between-group variation in mouse skin samples is significantly higher than the within-group variation, confirming a notable difference in microbial composition between the groups. ANOSIM (*R* = 0.2551, p-value = 0.009) further supports this finding, indicating a moderate but significant separation between the microbial communities across radiation doses (Supplemental Fig. 3). Beta diversity via Bray-Curtis Dissimilarity (ANOSIM, PERMANOVA, and Kruskal-Wallis tests, p-value < 0.05) was highest in the 1 Gy samples (0.724 ± 0.152), indicating greater variability in community composition in this group, possibly reflecting microbial adaptation to moderate radiation. The lower beta diversity in the 4 Gy and sham groups suggest more uniform microbial communities, either due to the stabilizing effects of no radiation (sham) or the selective pressures of high radiation (4 Gy). These findings highlight the dose-dependent effects of radiation on microbial diversity.

Differential taxonomic analyses were conducted at the level of species. Most significantly affected species (ANOVA, p-value < 0.05) included many undermined bacteria except Streptococcus and Staphylococcus in mouse skin at 1 Gy (Supplemental Table 4). However, most of them belonged to *Clostridia*, *Bacilli*, and *Gammaproteobacteria* classes in the skin in both mice and coHSE exposed to radiation although in different proportions (Fig. 5b). We observed a decreasing trend in *Clostridia* species in both coHSE and mouse skin at 4 Gy compared to 1 Gy, particularly in the ones belonging to the family of the *Lachnospiraceae*. Conversely, an increase in species belonging to the *Actinobacteria* and *Bacteroidia* classes were observed in in both coHSE and mouse skin at 4 Gy compared to 1 Gy.

## Discussion

This study evaluated a coHSE model as an innovative and long-lasting in vitro platform for studying the effects of radiation exposure on the skin. By incorporating natural mixed microbial colonization into a bioengineered human skin model, our research bridges significant gaps in existing radiobiological studies that rely primarily on animal models or 2D cultures. Time since radiation exposure is a critical criterion for patient triage in the scenario of a nuclear explosion. The Labskin model used in this study demonstrated robust culture stability for at least 25 days post-inoculation, including a 4-week post-irradiation period, supporting its utility in extended experimental timelines. While the combination of microbial colonization and subsequent irradiation posed potential challenges, this dual-stress paradigm had not been previously investigated to our knowledge. Despite these stresses, the model remained structurally and functionally competent throughout the experimental period. This was evidenced by consistent cell survival, preserved tissue integrity, and multi-omic signatures that closely align with known in vivo responses to radiation. These results support the suitability of the coHSE system as a durable and translational in vitro platform for investigating the effects of radiation on human skin.

The skin, as the first barrier exposed to radiation, represents a promising avenue for biodosimetry due to its accessibility and its role in systemic health. However, previous models, including rodent skin or 2D cultures, have inherent limitations, such as structural and microbiological differences from human skin. The colonized HSE model described in this study overcomes these barriers by providing a full-thickness skin equivalent capable of prolonged experimental designs while allowing microbial colonization. Our findings reinforce its potential to serve as a model of predilection for radiation research on human skin and the development of non-invasive radiomitigation measures.

Our results demonstrate that radiation exposure of up to 4 Gy, does not significantly impair the viability of coHSE or introduce opportunistic microbial overgrowth beyond manageable levels when simple procedural modifications are implemented. This validates the model’s suitability for extended experiments. Our findings also revealed that radiation exposure may induce alterations in the epidermal layer (Figs. 1c and 2a), potentially mimicking radiation-induced desquamation observed in humans^34^. To further evaluate tissue integrity, we assessed delamination scores, epidermis-to-dermis ratios, dermal cell density, and PCNA expression. These metrics are important indicators of morphological stability, which can directly influence swab sample yield consistency and the interpretability of downstream multi-omics analyses. In this context, the absence of statistically significant differences in delamination scores between 1, 2, and 4 Gy-exposed coHSEs indicates preserved tissue integrity across doses. Similarly, we observed no significant changes in epidermis/dermis ratios during the first week post-irradiation, nor in dermal cell density at weeks 1 and 4, suggesting minimal architectural disruption following photon exposure. This homogeneity ensures that such alterations do not introduce significant bias when assessing molecular and microbial profiles. This is a crucial observation, as it validates the reliability of the coHSE model for non-invasive sampling methods, such as swabbing, and subsequent molecular and microbial profiling, even in the presence of radiation damages such as skin desquamation.

Importantly, microbial inoculation has been shown to decrease cell proliferation and increase cell differentiation benefiting the skin barrier function^32,37,38^. Consistently, PCNA expression significantly declined over time at all radiation doses, indicating a time-dependent reduction in proliferative activity. However, this occurred without any measurable loss in dermal cell density which was maintained within naturally ranges (10^3^ cell.mm^−2^ in 1 mm thick skin)^39^ further supporting the structural resilience of the coHSE model. Collectively, these analyses demonstrate that coHSEs retain sufficient morphological stability post-irradiation to support reproducible, swab-based molecular investigations over time.

Dyslipidemia and impaired lipid metabolism are frequently reported events after radiation exposure in various tissues including the skin^40–47^. While substantial structural and physiological differences exist between mouse and human skin, such as thinner epidermis, higher hair follicle density, and differing immune and lipid profiles, we observed high overlapping lipidomic perturbations in both models following radiation exposure, with 60% of affected lipid subclasses being commonly found in both coHSE and mouse skin in response to radiation, which point out the existence of a well conserved lipidomic response to radiation and that robust biomarkers of radiation exposure might be found in the coHSE lipidome. Consistently, lipid metabolism was also prevalent in metabolomic analyses, with arachidonic acid (AA) metabolism and fatty acid metabolism being the most affected pathways in the skin in both models. Although murine skin is not directly homologous to human skin, the recurrence of these metabolic signatures accross species suggests the existence of conserved lipid remodeling in response to radiation and potential lipid bimarkers of radiation exposure in the skin. While acknowledging the limits inherent to interspecies extrapolation afore mentioned, we interpret these findings as cross-model molecular convergence that strengthens the plausibility of the mechanisms observed in the human-engineered tissue and its translational framework.

Mechanistically, radiation-induced enrichment of AA metabolism and associated glycerophosphocholine alterations observed in our study align with well-characterized inflammatory and cytoprotective lipid signaling pathways. Increased accumulation of lipids, especially unsaturated fatty acids, is reported as a pro-survival mechanism that can dampen radiation induced oxidative stress and DNA damge in glioblastoma^43^. As such, enriched fatty acid metabolism pathways in the skin after radiation may reflect an underlying protective mechanism, such as the accumulation of unsaturated fatty acids. The skin’s lipid bilayer, rich in phosphatidylcholine, is particularly susceptible to oxidative remodeling under ionizing radiation. Upon radiation exposure, cytosolic phospholipase A2 (cPLA2) is activated and hydrolyzes membrane phospholipids, especially phosphatidylcholine, which release AA and lysophosphatidylcholine (LPC)^48,49^. These lipid mediators act as potent signaling molecules, modulating inflammation, DNA damage response, cell viability, and tissue regeneration. Interestingly, AA release via cPLA2 is critical for maintaining viability of irradiated vascular endothelial cells through ERK1/2 and Akt signaling^50^. Consistently, we found increased expression of the proliferation marker PCNA with dose exposure levels and maintained cell density over time in all groups. Oxidative degradation of AA leads to the formation of reactive α,β-unsaturated aldehydes such as the 4-hydroxy-2-nonenal (4-HNE), a major product of n-6 fatty acid peroxidation capable of promoting both cell death and cell survival. 4-HNE is a highly reactive and genotoxic electrophile that can induce the formation of DNA adducts while inhibit nucleotide excision repair (NER) pathway proteins, thereby reducing cellular capacity to resolve radiation-induced DNA lesions^51^. However, due to its oxidant status, 4-HNE can also induce cellular defense mechanisms against oxidative stress, triggering its own detoxification thereby promoting cell survival^52^. In addition to its roles in inflammation and cyto-protection, AA has been shown to actively promote skin regeneration through the GPR40/ERK signaling axis in keratinocytes^53^. This suggests that radiation-induced release of AA may not only initiate inflammatory cascades but can also promote epidermal viability trough both cell survival and tissue repair mechanisms which are critical for maintaining skin barrier function after injury. As such, the AA and fatty acid oxidation metabolism pathways enrichment observed in our coHSE model may reflect a complex response to radiation-induced oxidative stress in the skin, including both inflammation processes and compensatory protective pathways that contribute to the increased cell survival and the preservation of tissue integrity following irradiation observed in our study. In both the coHSE and mouse models, the metabolomic and lipidomic profiles capturing these signatures may thus point to a coordinated response involving inflammation, tissue remodeling, and repair processes, all of which are relevant to both acute and delayed manifestations of radiation-induced skin toxicity. Collectively, these findings support the notion that AA pathway alterations may constitutes an early molecular indicator of radiation-induced skin remodeling with potential application for skin-based biodosimetry.

Overall, shared pathway enrichments, suggest conserved molecular responses, reinforcing the model’s translational applicability while the unshared observed in coHSEs may represents the missing part of the physiological picture uncaptured in mice models due to inherent structural and cellular differences between human and mouse skin as well as laboratory environmental pressures. Additionally, microbial diversity analyses revealed dose-dependent shifts in microbial composition, with most of species belonging to specific microbial classes, such as *Actinobacteria* and *Clostridia*^10,15,17,55^. Lachnospiraceae are commonly found on the skin and play a critical role in maintaining barrier integrity, primarly through the production of short-chain fatty acids (SCFAs) like butyrate and by directly competing with pathogenic organism^76,77^. Additionaly, *Cutibacterium* (elevated in Labskin 1 Gy and mouse 4 Gy groups) and *Corynebacterium* (elevated in Labskin 4 Gy), two lipophilic genera that are part of the natural skin microflora, were among the most upregulated taxa in both models. These bacteria possess lipase activity that allows them to hydrolyze triglycerides in sebum, releasingfree fatty acids and SCFAs, including monounsaturated fatty acids^78–80^. These lipid products help maintain a low skin pH, inhibiting the growthof pathogenic microorganisms^81,82^. Beyond their antimicrobial effects, free fatty acids also enhance innate skin immunity by simulating the expression of human β-defensin 2 (hBD-2), one of the most abundant antimicrobial peptides (AMPs) in human skin^83^. SCFAs, in particular, exert well-documented anti-inflammatory and immunomodulatory effects, supporting host resilience and reducing inflammation in epithelial tissues^84–86^. Furthermore, *Micrococcaceae*, a family of Gram-positive bacteria also part of the natural bacterial population on human skin^87^, are also amongst the most affected taxa in our study. Interestingly, members of the *Micrococcaceae* family are known for their radiation resistance, particularly some species found in skin like *Micrococcus* and *Kocuria* species^88–91^. In our dataset, *Kocuria *species were downregulated 3- to 4-fold in coHSE, and an unidentified Micrococcus species was consistently downregulated in both models. Collectively, these findings underscore the sensitivity of skin microbial communities to radiation exposure and suggest that both beneficial commensals (e.g., *Lachnospiraceae*, *Lactobacillaceae*) and radiation-resistant taxa (e.g., *Micrococcaceae*) undergo reproducible alterations after radiation exposure. These microbial changes likely influence host immune tone, barrier function, and tissue repair capacity. The consistent detection of similar taxonomic alterations in both coHSE and murine models reinforces the translational potential of the coHSE system as a platform to study radiation-induced microbiome remodeling and its implications for personalized risk stratification in exposed populations.

Thus, the coHSE is a promising model for radiation research including various applications, from biodosimetry to radiotherapy optimization and space radiation research. Approximately 95% of cancer patients undergoing radiotherapy develop some form of radiation-induced dermatitis, including erythema, ulceration, edema, pruritus, and desquamation^34^. These conditions increase pain and lead to complications including decreased self-esteem and image, stigma associated with radiation exposure, increased financial burden associated with treatment, impaired ability to complete daily activities, delay in treatment^35^. Additionally, increased skin cancer risk has been reported in various exposed groups, including bomb survivors^55^ uranium miners^56^as well as early 20th century radiologists^57^ with a risk up to 57% higher n radiotherapy treated breast cancers patients^58^. Better understanding of the molecular response to radiation of the skin is essential to mitigate radiation-induced skin injuries including elevated cancer risks, delays in treatment, diminished aesthetic appeal, and reduced quality of life. The coHSE system, as an ethical and scalable alternative to animal models, could also potentially greatly benefit the field of space research, where research payload size and mass are determinant funding criteria.

Despite the promising results and translational relevance of the coHSE model, several limitations must be acknowledged. First, although X-rays provide a biologically relevant and technically accessible surrogate to gamma rays, they do not replicate the full spectrum of radiation qualities encountered in mixed-field nuclear incidents or space environments, which often include high-linear energy transfer (high-LET) radiation such as protons, alpha particles, or heavy ions. Additionally, the photon energy used in this study (up to 320 kVp) represents a lower energy spectrum than that encountered in real-world scenarios such as nuclear detonations or solar particle events, potentially affecting depth dose distribution and tissue interaction. Furthermore, the 1–4 Gy dose range used, although clinically and operationally relevant, does not encompass ultra-low-dose chronic exposures or high-dose acute exposures (> 6 Gy) relevant to certain radiological emergencies or therapeutic settings. Future studies should expand to include a broader spectrum of radiation energies and qualities to fully evaluate the versatility and robustness of the coHSE to model physiological responses under diverse radiation exposure scenarios.

Receiving a substantial amount of the blood flow, it is very likely that skin conditions might reflect internal health status, as it is already well documented for cardiovascular conditions^4,8,59^. While the current system enables the coculture of the HSE insert with another cell type within the same well, future studies should also explore the combination of the coHSE model with other engineered systems to address the systemic limitation. Recently, a tissue-chip system has been developed which is fully ready-applicable, including human heart, liver, bone and a full thickness HSE linked by a circulating vascular flow allowing for the recapitulation of interdependent organ functions^60^. Although the system did not include a microbial component which is critical for a true recapitulation of skin physiology, it relied on a HSE insert system highly similar to the one presented in this study, suggesting straightforward compatibility and enabling immediate implementation for future studies. The field of bioengineering is continuously evolving to closely mirror the natural biological system. Labskin HSEs containing melanocytes are now available^61^. This represents an additional critical asset for the future of radiation research since melanocytes and melanin production are heavily studied to decipher their protective properties against radiation and develop biomimicking countermeasures^62^.

Overall, this study shows that the coHSE model represents a significant step forward in radiation biology research, closely recapitulating the biological mechanisms underlying the radiation effects in the skin, enabling a better understanding of radiation biology and the development of better tailored medical tools accordingly, while advancing ethical research practices. While skin research is relatively nascent in radiation biology, more attention and efforts should continue to fully characterize the molecular dynamism of the human skin in response to radiation, as it represents the most convenient platform for rapid biodosimetry. Skin studies and skin biomarker discovery will provide critical insights for the development of real-time biosensing and health status monitoring. We advocate for a systematic implementation of coHSE models as a standard experimental method in radiation studies involving the skin.

## Materials and methods

### HSE and Microbiome inoculation

HSE models were purchased from the Labskin UK, Deepverge company. The production method of this model can be found in Holland et al., 2008 ^22^. The Labskin model is a 3D bioengineered HSE with full layering thickness including both dermal and epidermal mimetics that can be additionally colonized by microbial inoculum and can be maintained in culture up to 25 days^22,63^. HSEs were shipped in solid medium at room temperature over 2 days. Upon arrival, HSE inserts were transferred into 12-well plates containing 1-mL medium and equilibrated at 37 °C, 5% CO_2_ overnight before micobial inoculation.

Human skin microbiome isolation from volunteers for transplantation onto human skin equivalent were carried out in accordance with relevant guidelines and regulations and was approved by Institutional Review Boards (Pro00067707). Informed consent was obtained from all participants. Microbiome isolates were collected from the external skin surface of both cheeks of the face of 38 volunteers recruited by American Type Culture Collection (ATCC) using a scrub-wash technique^64^. Isolates from both cheeks were pooled to yield a higher colony-forming unit (CFU). Donor volunteers were from various ages and ethnicities, with equal representation of sexes to truly represent human microbial diversity (Supplemental Tables 1 and 2). Before colonization, donor isolates were washed in sterile dH_2_O and centrifuged twice at maximum *g* for 10 min to remove residual detergent and other contaminants that could impair downstream analysis. Isolates were then inoculated to HSE at 1.10^4^ CFU/construct (10 µl at 1.10^6^ CFU/ml) one day prior to irradiation (age of HSE = 14 days). Eight HSEs were not inoculated for later comparisons. The day after, coHSE were exposed to x-rays (0 Gy *n* = 8, 1 Gy *n* = 6, 2 Gy *n* = 9, or 4 Gy *n* = 6). Skin swab samples were collected prior and up to 25 days after exposure. The skin swab sample collection was standardized and consisted of 10 gentle rounds, always performed in the same direction, over the surface of each HSE construct for a time duration of 10 s.

### Animal model

All animal procedures were approved by the Institutional Animal Care and Use Committee protocol file number 270,036 at Lawrence Berkeley National Lab (LBNL) with the OLAW assurance number D16-0031 (A3054-01). All methods were carried out in accordance with all relevant federal and state guidelines and are reported in accordance with ARRIVE guidelines^65^. C57BL/6J male and female mice (9–11 weeks old) were purchased from Jackson Laboratories (Sacramento, CA) and acclimated for two weeks before radiation exposure at doses 0 Gy or “sham” (*n* = 6), 1 Gy (*n* = 6), 2 Gy (*n* = 4), and 4 Gy (*n* = 6). Mice were shaved on a lateral area of 1.5 × 6 cm at least one day prior to swab collection to allow for recovery of the skin microbiota. Skin swab samples were collected 2 and 1 day prior to irradiation, and at 8 time points up to 25 days post exposure. Housing conditions were standardized, monitored, and recorded by animal facility staff. Mice were housed on P.J. Murphy’s Sani-Chips (P.J. Murphy Forest Products Corp.).

### Irradiation

For the coHSE model, irradiations were performed the day after inoculation with the XRAD 320 irradiator (320 kVp, 12.5 mA) fitted with a beam-conditioning filter (0.75 mm Tin + 0.25 mm Copper + 1.5 mm Aluminum) providing a specific beam Half Value Layer (HVL) of ≅ 4 mm Cu at 50 cm from the source. The dose rate was 0.85 Gy/min and dosimetry was performed using a NIST-traceable ionization chamber (Radcal 10 × 6-6, Monrovia CA) calibrated to air kerma. For mice, total body irradiation was performed in the XRAD 320 irradiator (300 keV/10 mA) fitted with a 0.5 mm Cu filter (HVL ≅ 3 mm Cu) and mice were placed on a rotating platform (4 RPM) at 50 cm from the source. The dose rate was 1.3 Gy/min. Dosimetry in the machine was performed with a RadCal ionization chamber (Radcal 10 × 6-0.18), as well as gafchromic film.

### Hematoxylin & eosin (H&E)

Detailed steps of the paraffin embedding process can be found in Supplemental Table 3. Briefly, HSEs were fixed in 10% (v/v) formalin for 24 h at room temperature and transferred to 70% ethanol. HSEs were then embedded in paraffin. The H&E protocol was adapted according to Labskin Deepverge methods. HSE sections of 5 μm were dehydrated with 3x xylene + 3 × 100% ethanol baths + tap water), and stained with Mayer hematoxylin (8 min), Scott’s bluing reagent (6 min), and Eosin Y 0.25% (1 min). Slides were then rehydrated (3 × 100% ethanol + 3x xylenes baths) and mounted with Cytoseal 60 (Epredia cat#23-244256).

### Cell proliferation measurement

A Ki67 immunostaining assay was used to measure cell proliferation in combination with K10 co-immunostaining to visualize the different skin layers. Sections were dehydrated in xylene, ethanol (100%, 90%, 70%) and dH_2_O baths. Antigen retrieval was performed using 0.01 M sodium citrate buffer (high pressure/temperature, pH 6) with 5% goat serum used as a blocking agent. Sections were incubated overnight in a humid chamber at 4 ˚C with a primary antibody Ki67 (1:100, Millipore #AB9260) and K10 (4 µg/ml, Invitrogen cat# MA1-06319). A goat anti-rabbit TRITC (1:1,000, abcam cat# ab6718) and a goat anti-mouse CY5 (1:200, Invitrogen cat# A10524) were used as secondary antibodies (1 h incubations at room temperature in darkness). Slides were mounted using a DAPI mounting media (Fluoromount-G, Invitrogen #50-112-8966). The number of Ki67 positive cells was divided by the total number of cells calculated by total nuclei (DAPI) to obtain a measure of the cell proliferation.

### Western blotting and quantification

Total protein was extracted from full layered coHSE using a RIPA protein extraction buffer supplemented with a protease/phosphatase-inhibitor cocktail (Thermo Scientific # PI78442). Total protein concentration was determined by the microBCA method (Thermoscientific MicroBCA Protein Assay Kit #23235). A total of 25 µg of protein per sample were electrophoresed on reducing sodium dodecyl sulfate polyacrylamide gels and transferred to PVDF membranes. After blocking with 10% goat serum in TBS-T buffer, membranes were incubated with a mouse PCNA primary antibody (ProteinTech #60097) in 3% BSA overnight at 4 °C. Following this, membranes were rinsed 3 times 15 min each with TBS-T buffer and then incubated with goat anti-mouse horseradish peroxidase (HRP)-conjugated secondary antibody (Cell Signaling # 14709 S) for 1 h at RT in 5% non-fat milk. The antibody complexes were detected using enhanced chemiluminescence reagents (Cytiva RPN2235) and labeled proteins were detected with the imaging Amersham 600 imager system. Protein expression was quantified using the Image lab software (Bio-Rad Laboratories) and normalized to total protein stained by amido black^66,67^.

### Untargeted metabolomic profiling

Skin swab samples were collected from mice or coHSEs, snap frozen in liquid nitrogen and stored at −80˚C in 1.5 mL tubes until further processing. Metabolite extraction was performed according to previous metabolomic analysis from swabs^7^. Each sample swab was immersed in 0.5 mL of 50%:50% ethanol: water containing internal standards (30 µM 4-nitrobenzoic acid, Sigma cat# 72910; 2 µM debrisoquine sulfate Sigma cat# D1306; 5 µM chlorpropamide Sigma cat# C1290 final concentrations) for 2 h at 4 °C. Samples were transferred to a new clean 1.5 mL tube and then dried in a speed vac with no heat. Dried samples were resuspended in 50 µl of water with 0.1% formic acid (FA). As needed, samples were filtered with a 0.2 μm filter (Bio-Inert^®^ Membrane 0.2 μm, aqua Cat# ODM02C34). Samples were then transferred to 250 µl vials (Thermo fisher #C4011-13) for subsequent liquid chromatography (LC) time-of-flight MS analysis. A quality control sample (QC) was constructed by pooling 5 µl of each sample.

LC-MS analysis was conducted with a protocol adapted from Afghani et al., 2021 ^7^. Samples were injected (1 µL) into a Waters Acquity Ultra Performance Liquid Chromatography (UPLC) with a BEH C18 1.7 μm, 2.1 × 100 mm column and coupled to a Xevo^®^ G2-S quadrupole time-of-flight (QTOF) MS (Waters, Milford, MA, USA). Positive and negative electrospray ionization (ESI) data-independent modes were used for data acquisition with leucine enkephalin ([M + H] + = 556.2771, [M-H]− = 554.2615) as Lock- Spray^®^. Operating conditions for ESI were: capillary voltage 2.75 kV, cone voltage 30 V, desolvation temperature 500 ◦C, desolvation gas flow 1000 L/Hr. Mobile phases consisted of the following: solvent A (water/0.1% FA) and solvent B (acetonitrile/0.1% FA). The gradient was set to 5% B for 1.12 min, followed by an increasing proportion of B to 99.5% at minute 6.4 and a plateau for the remaining 3.6 min. Column temperature was kept at 40 °C, and flow rate was set to 0.4 mL/min. Blanks and QC samples were run every 10 samples.

### Targeted lipidomic profiling

Skin swab samples were collected from mice or coHSEs, snap frozen in liquid nitrogen and stored at −80 °C in 1.5 ml tubes until further processing. Lipid extraction was performed according to previous lipidomic analyses of swab sample methods^68^. Each sample swab was immersed in 0.5 mL of 70%:30% ethanol: water containing lipid standards (1 µg/ml EquiSPLASH^®^ LIPIDOMIX, Avanti cat# 330731-1EA; 2.5 µg/ml 18:1 Chol (D7) ester Avanti cat# 700185 M-1MG; 5 µg/ml 15:0–18:1-d7-PA Avanti cat# 791642 C-1MG) for 2 h at 4˚C. Samples were transferred to a new clean 1.5 ml siliconized tube (CPLab Safety, cat# BP-4167SLS50) and 10 µl of samples were saved and stored at −80 °C for protein quantification. One ml of chloroform/methanol/water (1:2:0.8) was added to each sample. Samples were centrifuged for 10 min at maximum speed at 4 °C, and the lower phase was placed in a new fresh 1.5 ml siliconized tube. Samples were dried in a speed vac with no heat and resuspended in 200 µl of methanol/isopropanol/acetonitrile (9.65v/1v/3v). As needed, samples were filtered with a 0.2 μm filter (Bio-Inert^®^ Membrane 0.2 μm, aqua Cat# ODM02C34). Samples were then transferred to 250 µl vials (Thermo fisher #C4011-13) for MS analysis (SCIEX 5500 QTRAP). A QC sample was constructed by pooling 5 µl of each sample together and injected every 10 samples along with blanks.

We injected samples (5 µL) onto a Xbridge amide column (3.5 μm, 4.6 × 100 mm) (Waters, Milford, MA, USA) maintained at 35 ^◦^C before analysis with a Sciex QTRAP 5500 Mass Spectrometer (Sciex, Framingham, MA, USA). Lipids were detected by multiple reaction monitoring (MRM) transitions in both positive and negative ionization modes (listed in Supplementary File S1). Mobile phases consisted of the following: solvent C (95% acetonitrile/5% water with 10 mM ammonium acetate) and solvent D (50% acetonitrile/50% water with 10 mM ammonium acetate). The gradient for solvent C and D were as follow: initial gradient 100% C, 3.0 min 99.9% C, 3.0 min 94% C, 4.0 min 25% C, 6.0 min 0% C, 6.0 min equilibrate back to 100% C, at a flow rate of 0.7 mL/min. Source and gas setting were as follows: temperature = 550 ◦C, nebulizing gas = 50 and heater gas = 60, curtain gas = 30, CAD gas = medium, ion spray voltage = 5.5 kV in positive mode and − 4.5 kV in negative mode. Blanks and QC samples were run after every 10 samples. We used a targeted lipid profiling assay designed to analyze several lipid groups including: cholesteryl esters (CE), cholesterol, ceramides (Cer), hexosylceramides (HexCer), lactosylceramides (LCER), dihydroceramides (DCER), sphingomyelins (SM), acylcarnitines, diacylglycerides (DAG), triacylglycerides (TAG), monoacylglycerides (MAG), free fatty acids (FFA), phosphatidic acids (PA), lysophosphatidic acids (LPA), phosphatidylcholines (PC), ether-linked phosphatidylcholines (ePC), lysophosphatidylcholines (LPC), phosphatidylinositols (PI), lysophosphatidylinositols (LPI), phosphatidylethanolamines (PE), ether-linked phosphatidylethanolamines (ePE), lysophosphatidylethanolamines (LPE), phosphatidylglycerols (PG), and phosphatidylserine (PS)^44^.

### Microbiome profiling (metagenomics)

Skin swab samples were collected from mice or coHSEs, snap frozen, and stored in stabilization buffer (Zymo Research Corporation DNA/RNA Shield, 250 ml, Fisher scientific #50-125-1706) at −80 °C in 1.5 ml tubes until further processing. Frozen samples were shipped on dry ice to Novogene and Azenta for 16 S and ITS sequencing.

### Data processing, statistical analysis

#### Software

Untargeted metabolomic data were processed with the Progenesis QI software (Nonlinear Dynamics, Newcastle, U.K.) for peak alignment and peak picking. Adducts for compound deconvolution were set to M + H, M + H–H_2_O, M + NH_4_, M + Na (ESI^+^) or M-H, M-H_2_O–H (ESI^−^). Data normalization was performed using software chosen QC chromatogram as alignment and normalization references applying a log-ratio scale factors calculated from all detected compounds in each sample (“normalize to all compounds” function). Putative identifications for spectral features were assigned using monoisotopic mass (± 8 ppm error), and theoretical fragmentation patterns from the human metabolome database (HMDB), LIPID MAPS, and the METLIN MS/MS empirical library^69–72^. For targeted lipidomics, raw data and peak areas were visually inspected using the MultiQuant v.2.0 software (Sciex, Framingham, MA, USA) and data were then exported to Microsoft Excel. Lipids present in the QC sample with a coefficient of variation > 25% were removed and not considered further.

Statistical tests, including principal component analysis (PCA), partial least square discriminant analysis (PLS-DA), ANOVA, and clustering, were run on MetaboAnalyst 6.0 online software (Statistical Analysis module) to identify discriminating features among metabolomic and lipidomic data^73^. Over representation analyses (ORA) were also conducted in MetaboAnalyst 6.0 (Enrichment Analysis module) using the Small Molecule Pathway Database (SMPDB) and the Kyoto Encyclopedia of Genes and Genomes (KEGG) databases to analyze enriched pathways in metabolomic data. ORA of enriched lipid subclasses were conducted for lipidomic using a library containing 1,250 sub chemical class metabolite sets or lipid sets.

For microbiome analyses, 16 S data was analyzed using the QIIME2 software to assign taxonomy and determine Alpha Diversity (Shannon and Simpson Indexes) and Beta Diversity (Bray-Curtis Dissimilarity) of irradiated coHSE and mouse skin^74^. Taxa with a constant or single value across samples were deleted, and near-constant variables were further filtered by an interquartile range variance filter of 40%. Differential taxonomy analysis was performed using the R DESeq2 package version 6.1.1.

#### Statistical analysis

All analyses were performed using GraphPad Prism 10.0 (GraphPad). The Mann-Whitney and Kruskal-Wallis nonparametric tests were used to compare two or more non-normally distributed groups. ANOSIM (R) and PERMANOVA (Pseudo-F) statistical tests were also run on beta diversity results using QUIME2. For all statistical tests, a significance level inferior to 5% (*P* < 0.05) was considered statistically significant and data were presented with the standard error of the mean (SEM).

## Supplementary Information

Below is the link to the electronic supplementary material.

Supplementary Material 1

## Data Availability

Raw 16 S rRNA sequencing data have been deposited in the NCBI Sequence Read Archive under accession number [PRJNA1308618] and are available at < https://www.ncbi.nlm.nih.gov/bioproject/1308618>. All other data supporting the findings are available from the corresponding author upon reasonable request.
