# Supplementary material for: Using a full thickness bioengineered human skin equivalent as a model for radiation biology research
Source: Sci Rep. 2025 Oct 6;15:34702. doi: 10.1038/s41598-025-17153-4 (PMC12501070; doi:10.1038/s41598-025-17153-4)
Supplement: Supplementary file 1 — Supplementary Material 1 [file 41598_2025_17153_MOESM1_ESM.docx]

**Using a full thickness bioengineered human skin equivalent as a model for radiation biology research**

**Supplemental material**

**Supplemental Table 1.** ATCC participant metadata

|  | Age range | | |
| --- | --- | --- | --- |
|  | 20- 40 years | 40 - 60 years | Total |
| Females | 8 (21%) | 15 (39%) | 23 (60%) |
| Males | 9 (24%) | 6 (16%) | 15 (40%) |
| Total | 17 (45%) | 21 (55%) | 38 (100%) |

**Supplemental Table 2.** ATCC participant metadata summary

| **Processing Sequence** | **Reagent** | **Duration (min.)** | **Temperature (^o^C)** | **Drain Time (sec.)** | **Delay** |
| --- | --- | --- | --- | --- | --- |
| **0** | 70% Ethanol | 0:55:00 | RT | 120 | Yes |
| **1** | 80% Ethanol | 0:55:00 | RT | 120 | No |
| **2** | 95% Ethanol | 0:55:00 | RT | 120 | No |
| **3** | 95% Ethanol | 0:60:00 | RT | 120 | No |
| **4** | Absolute Ethanol | 0:55:00 | RT | 120 | No |
| **5** | Absolute Ethanol | 0:60:00 | RT | 120 | No |
| **6** | Clear Rite | 0:55:00 | RT | 120 | No |
| **7** | Clear Rite | 0:55:00 | RT | 120 | No |
| **8** | Clear Rite | 0:60:00 | RT | 120 | No |
| **9** | Paraffin Wax | 0:60:00 | 60 | 140 | No |
| **10** | Paraffin Wax | 0:60:00 | 60 | 140 | No |
| **11** | Paraffin Wax | 0:60:00 | 60 | 140 | No |

**Supplemental Table 3.** Paraffin embedding protocol. *RT: Room Temperature

**Java script for automated cell count in Qpath adapted from** [**https://qupath.readthedocs.io/en/0.4/docs/deep/stardist.html**](https://qupath.readthedocs.io/en/0.4/docs/deep/stardist.html)

*setImageType('FLUORESCENCE');*

*import qupath.ext.stardist.StarDist2D*

*createAnnotationsFromPixelClassifier("EpidermisDermis", 0.0, 0.0, "INCLUDE_IGNORED", "SELECT_NEW")*

*// Specify the model file (you will need to change this!)*

*def pathModel = '/Users/Geraldine/Downloads/dsb2018_heavy_augment.pb'*

*def stardist = StarDist2D.builder(pathModel)*

*.threshold(0.5) // Probability (detection) threshold*

*.channels('DAPI') // Specify detection channel*

*.normalizePercentiles(1, 99) // Percentile normalization*

*.pixelSize(0.5) // Resolution for detection*

*.measureShape() // Add shape measurements*

*.measureIntensity() // Add cell measurements*

*.build()*

*// Run detection for the selected objects*

*def imageData = getCurrentImageData()*

*def pathObjects = getSelectedObjects()*

*if (pathObjects.isEmpty()) {*

*Dialogs.showErrorMessage("StarDist", "Please select a parent object!")*

*return*

*}*

*stardist.detectObjects(imageData, pathObjects)*

*println 'Done!'*

*runObjectClassifier("Ki67 classifier");*

**Supplemental Figure 1.** A) Timeline of the procedures in air/liquid interface (ALI) days, B) HSE cell density at day of shipping (day ALI 12), and C) 24h post-inoculation (day ALI 16), D) coHSE dermis cell proliferation rate at 1, 3 and 7 days post-irradiation (day ALI 17, 19, and 23), and E) Western blot membrane pictures of PCNA relative expression in protein samples extracted from 1, 2 and 4 Gy x-rays exposed coHSE over 4 weeks. Full images of blots in Supplemental Figures 4 & 5

**
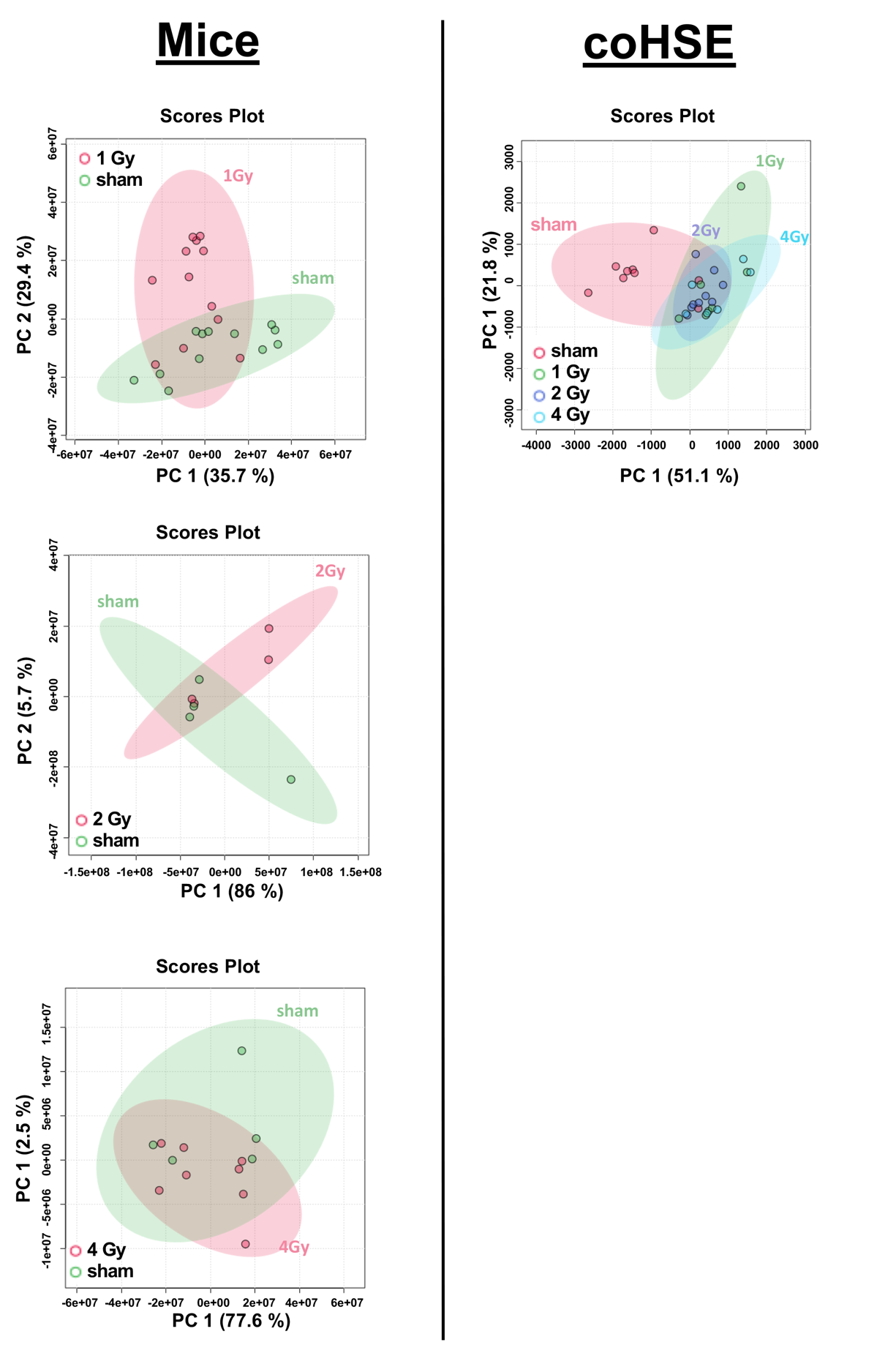
Supplemental Figure 2.** PCA of metabolomic spectral features in mice skin (left) and in coHSE (right) exposed to sham, 1, 2 and 4 Gy of x-rays

**
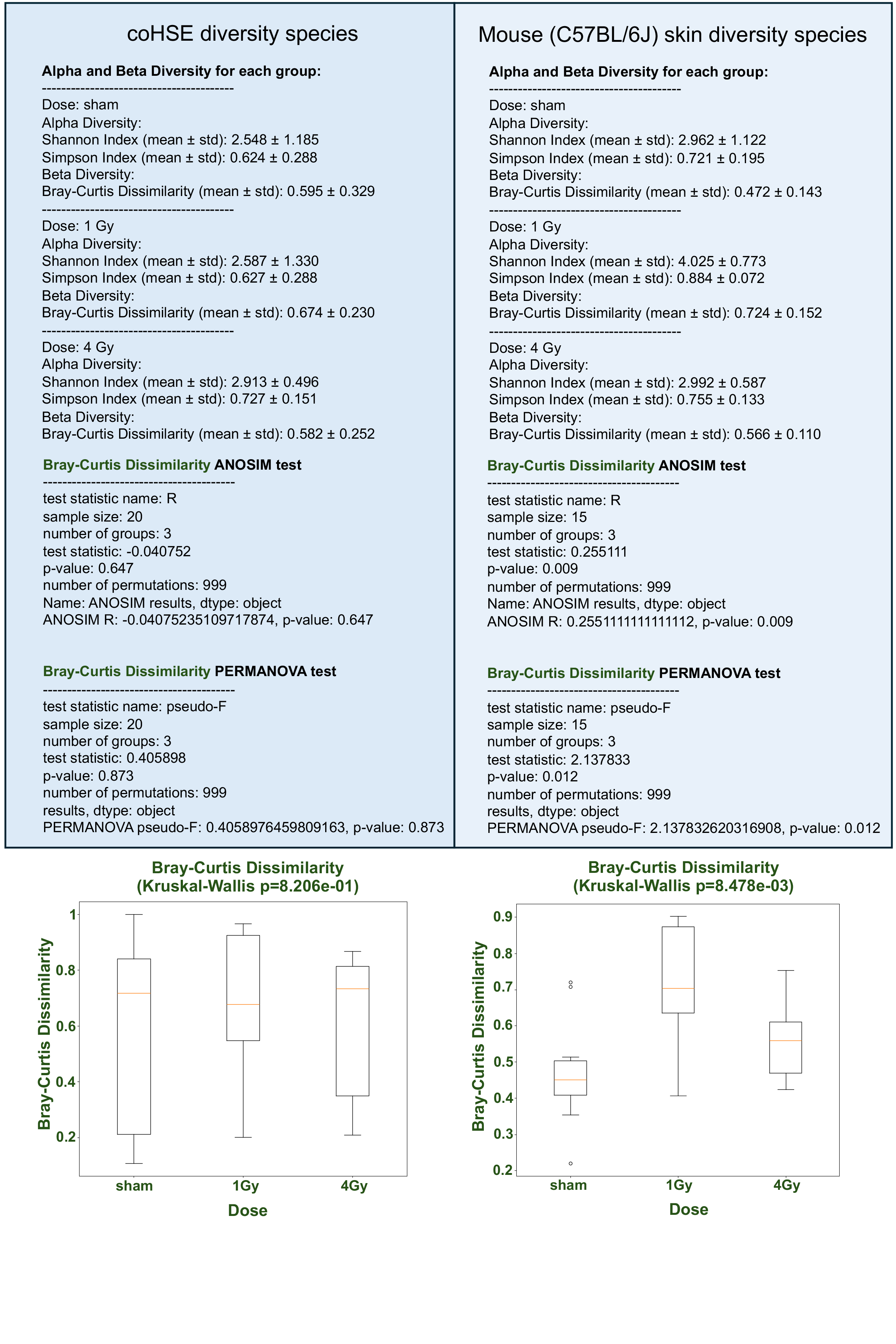
Supplemental Figure 3.** Alpha and beta microbial diversity in coHSEs and mouse skin exposed to Sham, 1 and 4 Gy of x-rays. A) Alpha and beta microbial diversity test results and B) Bray-Curtis Dissimilarity Kruskal-Wallis in coHSEs (left) and mouse skin (right).

**Supplemental Table 4.** Differential analysis at species level


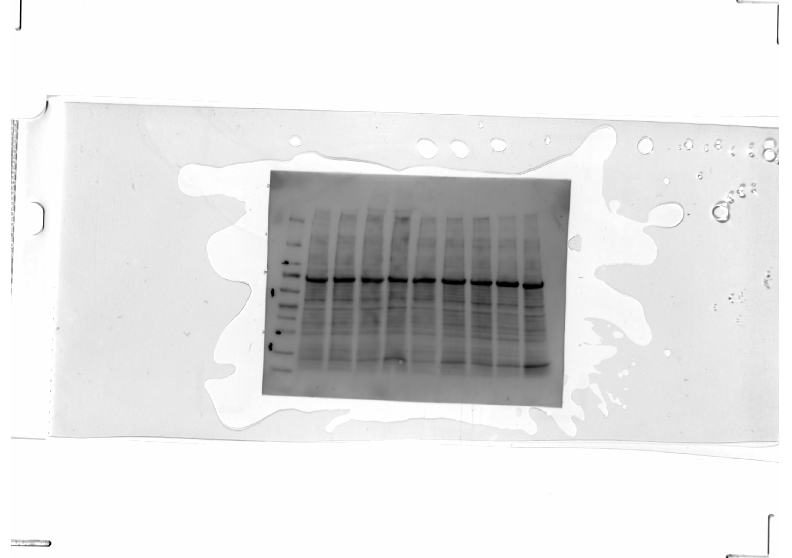

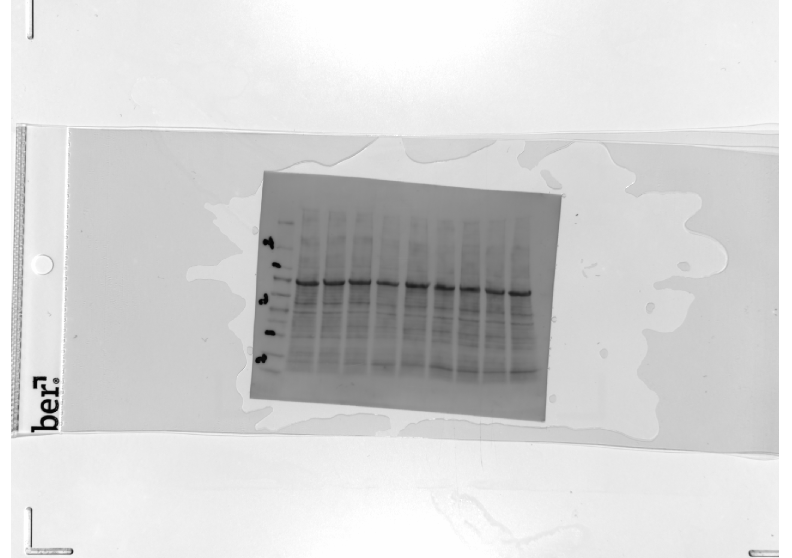

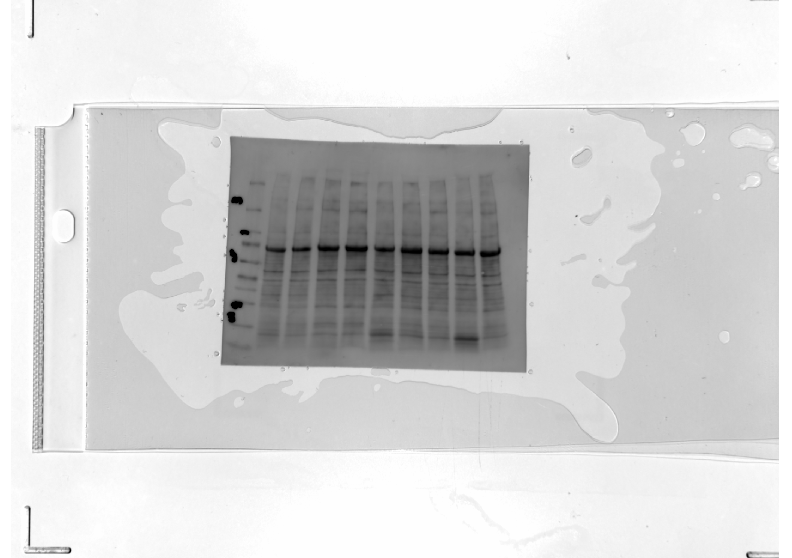

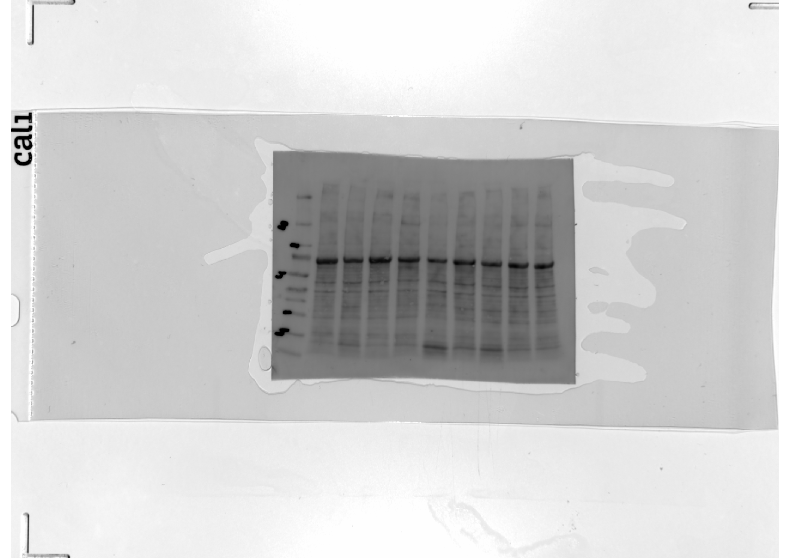

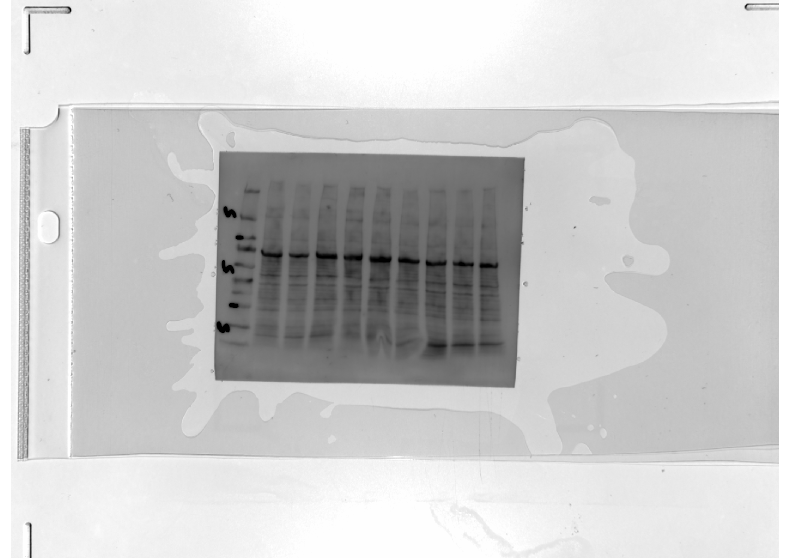

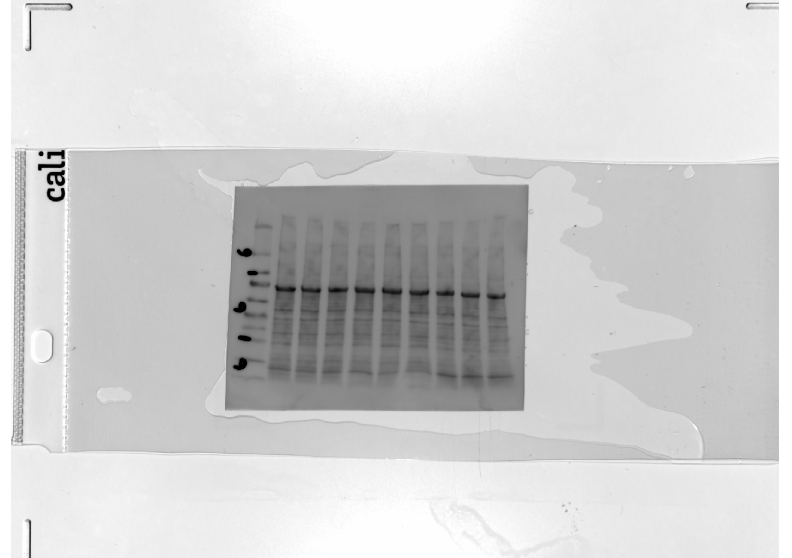


**1 Gy**

**2 Gy**

**4 Gy**

**R1**

**R2**

**R3**

**R1**

**R2**

**R3**

**R1**

**R2**

**R3**

**1^st^ week**

**2^nd^ week**

**4^th^ week**

**1^st^ week**

**2^nd^ week**

**4^th^ week**

**Ladder**

**R1**

**R2**

**R3**

**R1**

**R2**

**R3**

**R1**

**R2**

**R3**

**Ladder**

**1^st^ week**

**2^nd^ week**

**4^th^ week**

**R1**

**R2**

**R3**

**R1**

**R2**

**R3**

**R1**

**R2**

**R3**

**Ladder**

**1^st^ week**

**2^nd^ week**

**4^th^ week**

**R4**

**R5**

**R6**

**R4**

**R5**

**R6**

**R1**

**R4**

**R5**

**Ladder**

**R3**

**R4**

**R5**

**R4**

**R5**

**R6**

**R4**

**R5**

**R6**

**Ladder**

**1^st^ week**

**2^nd^ week**

**4^th^ week**

**R3**

**R4**

**R5**

**R4**

**R5**

**R6**

**R4**

**R5**

**R6**

**Ladder**

**1^st^ week**

**2^nd^ week**

**4^th^ week**

**Blots 1**

**Blots 2**

**Supplemental Figure 4.** Full images of blots #1 and #2 stained with amidoblack to reveal all proteins in each sample. R1, R2, R3, R4, R5, R6 indicate single replicate names for replicate 1, replicate 2, replicate 3, replicate 4, replicate 5, and replicate 6 respectively for each dose, each week. Note that 1 Gy 4^th^ week replicate 1 (R1), 2 Gy 1^st^ week replicate 3 (R3), and 4 Gy 1^st^ week replicate 3 (R3) are used in both blots 1 and blots 2 for inter-blot protein normalization, while total protein levels (amidoblack intensity) are used for intra-blot protein normalization.


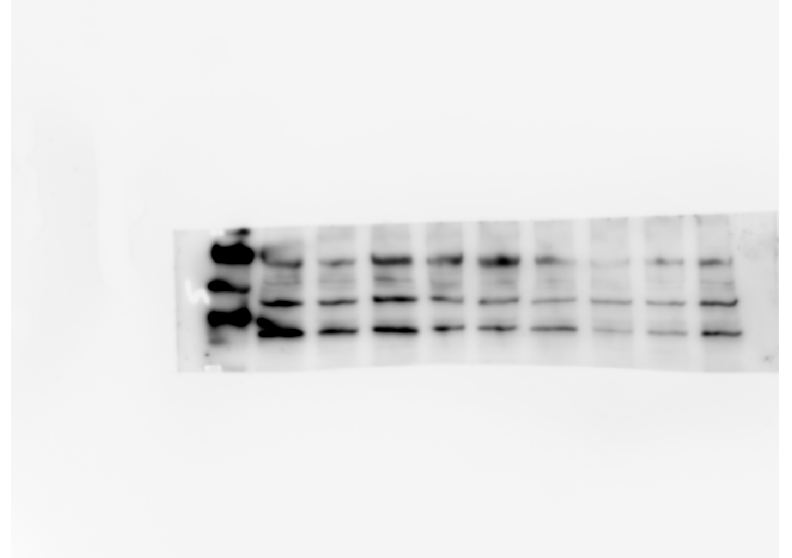

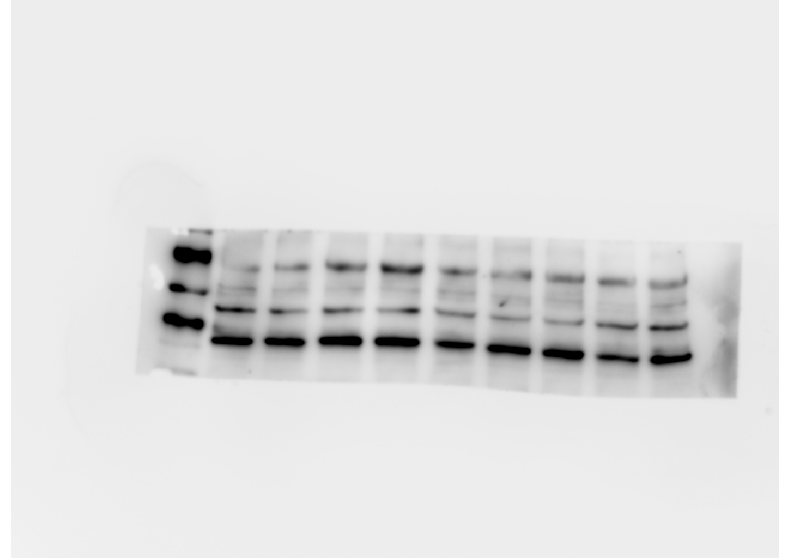

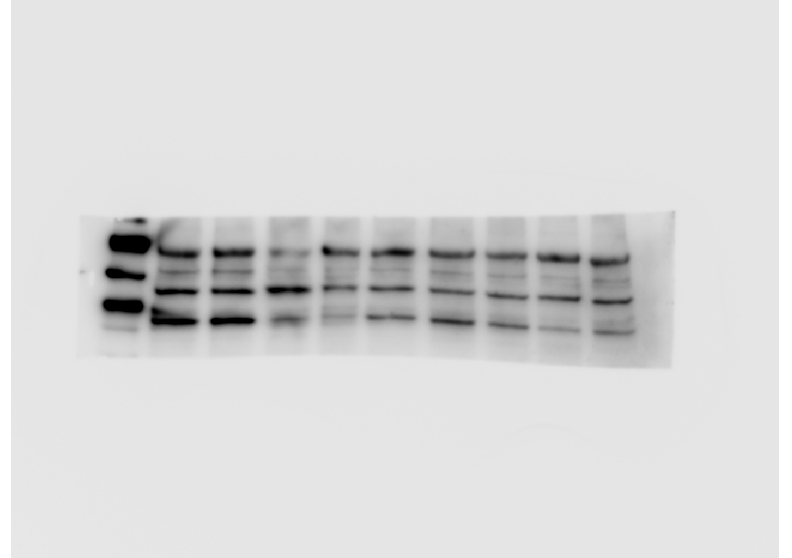


**PCNA 36-38 kDa, (Proteintech #60097)**

**PCNA 36-38 kDa, (Proteintech #60097)**

**PCNA 36-38 kDa, (Proteintech #60097)**

**R3**

**R2**

**R1**

**R3**

**R2**

**R1**

**R3**

**R2**

**R1**

**R3**

**R2**

**R1**

**R3**

**R2**

**R1**

**R3**

**R2**

**R1**

**R3**

**R2**

**R1**

**R3**

**R2**

**R1**

**R3**

**R2**

**R1**

**4^th^ week**

**2^nd^ week**

**1^st^ week**

**Ladder**

**100 kDa**

**70 kDa**

**50 kDa**

**40 kDa**

**35 kDa**

**25 kDa**

**4^th^ week**

**2^nd^ week**

**1^st^ week**

**Ladder**

**100 kDa**

**70 kDa**

**50 kDa**

**40 kDa**

**35 kDa**

**25 kDa**

**4^th^ week**

**2^nd^ week**

**1^st^ week**

**Ladder**

**100 kDa**

**70 kDa**

**50 kDa**

**40 kDa**

**35 kDa**

**25 kDa**

**Supplemental Figure 5a.** Full images of blots #1 from PCNA Western Blot using an antibody anti-PCNA from Proteintech #60097 R1, R2, and R3, indicate single replicate names for each dose, each week. Amidoblack whole protein staining (Supplemental figure 5) is used for protein normalization within each blot (Ri PCNA intensity/amidoblack intensity), while 1 Gy 4^th^ week replicate 1 (R1), 2 Gy 1^st^ week replicate 3 (R3), and 4 Gy 1^st^ week replicate 3 (R3) are used to normalize protein expression between blot 1 et 2 of each dose respectively (ex 1 Gy blots: Ri PCNA x [1^st^ week R3 PCNA blot1/1^st^ week R3 PCNA blot2])

**4 Gy**

**Blot 1**

**2 Gy**

**Blot 1**

**1 Gy**

**Blot 1**


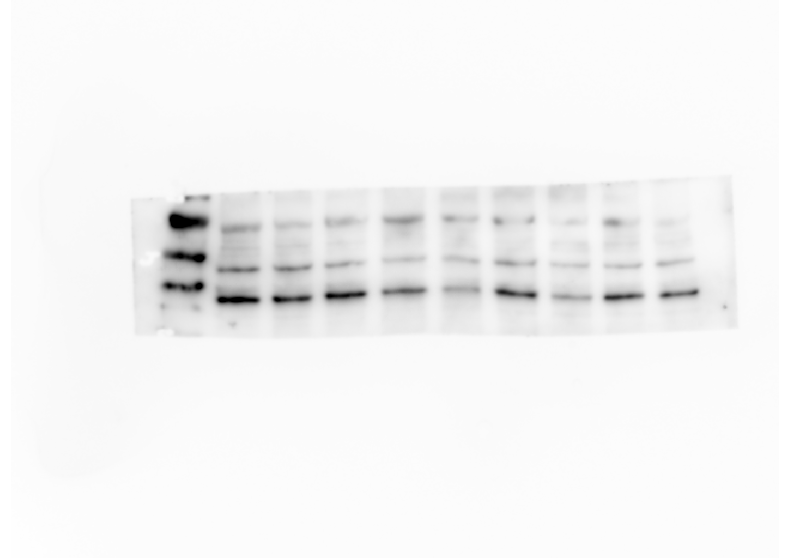

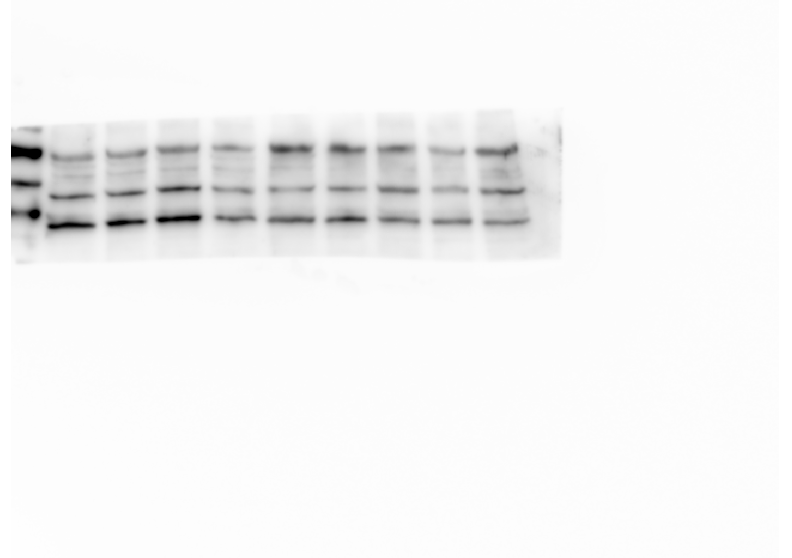

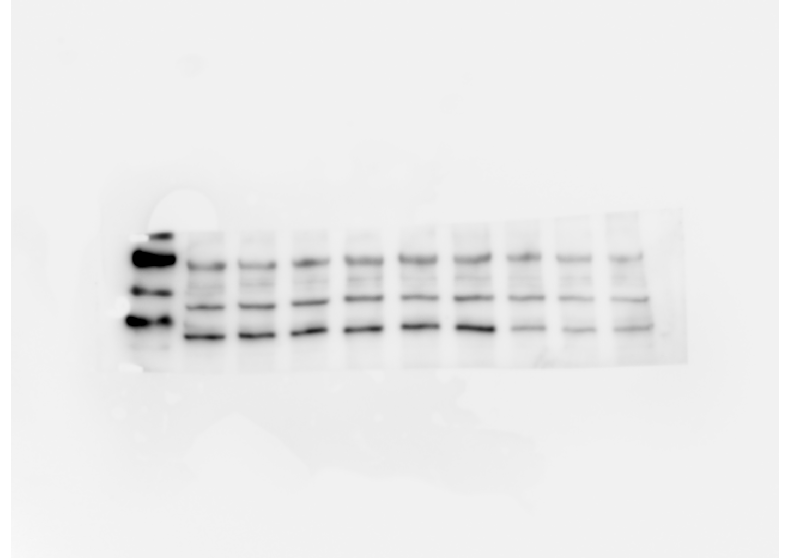


**Supplemental Figure 5b.** Full images of blots #2 from PCNA Western Blot using an antibody anti-PCNA from Proteintech #60097. R1, R3, R4, R5, R6 indicate single replicate names for each dose, each week. Amidoblack whole protein staining is used for protein normalization within each blot (Ri PCNA intensity/amidoblack intensity), while 1 Gy 4^th^ week replicate 1 (R1), 2 Gy 1^st^ week replicate 3 (R3), and 4 Gy 1^st^ week replicate 3 (R3) are used to normalize protein expression between blot 1 et 2 of each dose respectively (ex 1 Gy blots: Ri PCNA x [1^st^ week R3 PCNA blot1/1^st^ week R3 PCNA blot2])

**R4**

**R4**

**R1**

**4 Gy**

**Blot 2**

**2 Gy**

**Blot 2**

**1 Gy**

**Blot 2**

**PCNA 36-38 kDa, (Proteintech #60097)**

**PCNA 36-38 kDa, (Proteintech #60097)**

**PCNA 36-38 kDa, (Proteintech #60097)**

**R6**

**R5**

**R6**

**R5**

**R4**

**R5**

**R4**

**R3**

**R6**

**R5**

**R6**

**R5**

**R4**

**R5**

**R4**

**R3**

**R5**

**R4**

**R6**

**R5**

**R4**

**R6**

**R5**

**R4**

**4^th^ week**

**2^nd^ week**

**1^st^ week**

**Ladder**

**100 kDa**

**70 kDa**

**50 kDa**

**40 kDa**

**35 kDa**

**25 kDa**

**4^th^ week**

**2^nd^ week**

**1^st^ week**

**Ladder**

**100 kDa**

**70 kDa**

**50 kDa**

**40 kDa**

**35 kDa**

**25 kDa**

**4^th^ week**

**2^nd^ week**

**1^st^ week**

**Ladder**

**100 kDa**

**70 kDa**

**50 kDa**

**40 kDa**

**35 kDa**

**25 kDa**
